# Cancer Control at the District Hospital Level in Sub-Saharan Africa: An Educational and Resource Needs Assessment of General Practitioners

**DOI:** 10.1200/JGO.18.00126

**Published:** 2019-01-22

**Authors:** Allison N. Martin, Kelly-Mariella Kaneza, Amol Kulkarni, Pacifique Mugenzi, Rahel Ghebre, David Ntirushwa, André M. Ilbawi, Lydia E. Pace, Ainhoa Costas-Chavarri

**Affiliations:** ^1^ University of Virginia, Charlottesville, VA; ^2^ University of Rwanda College of Medicine and Health Sciences, Kigali, Rwanda; ^3^ Rwanda Military Hospital, Kigali, Rwanda; ^4^ University of Minnesota Medical School, Minneapolis, MN; ^5^ Yale School of Medicine, New Haven, CT; ^6^ MD Anderson Cancer Center, Houston, TX; ^7^ Brigham and Women’s Hospital, Boston, MA; ^8^ Boston Children’s Hospital, Boston, MA

## Abstract

**PURPOSE:**

The WHO framework for early cancer diagnosis highlights the need to improve health care capacity among primary care providers. In Rwanda, general practitioners (GPs) at district hospitals (DHs) play key roles in diagnosing, initiating management, and referring suspected patients with cancer. We sought to ascertain educational and resource needs of GPs to provide a blueprint that can inform future early cancer diagnosis capacity–building efforts.

**METHODS:**

We administered a cross-sectional survey study to GPs practicing in 42 Rwandan DHs to assess gaps in cancer-focused knowledge, skills, and resources, as well as delays in the referral process. Responses were aggregated and descriptive analysis was performed to identify trends.

**RESULTS:**

Survey response rate was 76% (73 of 96 GPs). Most responders were 25 to 29 years of age (n = 64 [88%]) and 100% had been practicing between 3 and 12 months. Significant gaps in cancer knowledge and physical exam skills were identified—88% of respondents were comfortable performing breast exams, but less than 10 (15%) GPs reported confidence in performing pelvic exams. The main educational resource requested by responders (n = 59 [81%]) was algorithms to guide clinical decision-making. Gaps in resource availability were identified, with only 39% of responders reporting breast ultrasound availability and 5.8% reporting core needle biopsy availability in DHs. Radiology and pathology resources were limited, with 52 (71%) reporting no availability of pathology services at the DH level.

**CONCLUSION:**

The current study reveals significant basic oncologic educational and resource gaps in Rwanda, such as physical examination skills and diagnostic tools. Capacity building for GPs in low- and middle-income countries should be a core component of national cancer control plans to improve accurate and timely diagnosis of cancer. Continuing professional development activities should address and focus on context-specific educational gaps, resource availability, and referral practice guidelines.

## INTRODUCTION

Incidence rates and mortality for many cancers are on the rise in low- and middle-income countries (LMICs), which contrasts with declining rates seen in many high-income countries.^1^ Much of the decline in cancer-related mortality observed in high-income countries has been attributed to public health programs, such as screening, early detection, and cancer education programs.^2^ In LMICs, these programs have not been fully developed and delays in care are common, generally falling into three categories: patient delays, provider delays, and system delays. Patient delays typically occur during the time between symptom onset or recognition and first contact with the health care system. Provider delays occur between the time of first contact with the health care system and the initiation of diagnostic work-up. System delays occur during the time between diagnostic work-up and the beginning of treatment.^3^ A growing body of evidence demonstrates that mitigating delays for common cancers, such as breast and colorectal cancer, can improve outcomes.^4,5^ Previous work from WHO has suggested that provider delays can be reduced via educational training, which can be provided through continuing professional development (CPD) courses.^6^ CPD courses are essential for improving provider capacity at the point of first contact. A needs assessment is an important first step in determining what skills and knowledge are most needed by these first-line providers. After the needs assessment, targeted CPD courses or a series of trainings can be developed as appropriate.

Rwanda is a densely populated, landlocked country in east Africa, with 60% of the population younger than age 24 years and nearly 70% of the population residing in rural areas as of 2017.^7^ The Rwandan health care system is structured to include lay community health workers at the front lines, generally followed by rural, nurse-staffed health centers, which are the point of first contact with the health care system for most Rwandans. The next level of care consists of district hospitals (DHs), which are staffed by general practitioners (GPs) who have completed 6 years of undergraduate medical education and a 1-year clinical internship, but who have not had specialized postgraduate medical training. The majority of physicians who practice in Rwanda are GPs. As the point of first contact for patients who present at the DH level, GPs are responsible for identifying and diagnosing patients with cancer, including the initial diagnostic work-up, such as pelvic and breast exams. They are then responsible for referral to higher-level hospitals for diagnostic confirmation via additional imaging and biopsies and definitive treatment with surgery or endoscopy.^8^ Whereas many cancer control efforts in Rwanda and other LMICs are aimed at the community level and primary health care centers, few efforts have so far been geared toward GPs who practice at DHs.

The primary aim of this study was to perform a subjective needs assessment in survey format to identify cancer-specific educational gaps among Rwandan GPs to develop educational objectives for future CPD courses. The secondary aim was to assess and describe Rwandan GPs’ perceptions of patient-, provider-, and system-level delays in cancer diagnosis and care to target these delays in future CPDs.

## METHODS

### Participant Recruitment

Ninety-six first-year GPs currently practicing at the DH level in Rwanda attended one of several 5-day CPD courses in early 2017 organized by the College of Medicine and Health Sciences of the University of Rwanda (CMHS-UR). The training course did not address any issues related to cancer diagnosis or treatment. Any GP stationed at any of the 42 DH locations in Rwanda and who attended the training was eligible for inclusion in the study. The GPs were offered the opportunity to participate in the study and each provided written informed consent. We administered surveys in either paper form or via QuestionPro, an online, Web-based electronic survey platform. QuestionPro is secured using multiple types of encryption, firewalls, and system monitoring mechanisms, and all QuestionPro employees have completed Health Information Portability and Accountability Act–compliance training.

### Needs Assessment Content

The cross-sectional needs assessment survey was designed to collect information in multiple domains, including detailed demographic information. To design the survey, we solicited input from multiple key informants, including current undergraduate medical students, GPs, and specialist physicians. In addition, we reviewed currently available educational materials used in the CMHS-UR curriculum for cancer diagnosis or treatment. The survey included questions that assessed issues related to patient delays (ie, financial barriers to seeking care and knowledge of cancer-concerning symptoms), provider delays (ie, practitioner physical exam skills and educational needs of GPs), and system delays at the DH level (ie, issues with patient transfer and referral, availability of medical technology, and availability of radiologic and pathology resources; Data Supplement).

### Ethics

This study was reviewed and approved by institutional review boards at both CMHS-UR and the University of Virginia. The informed consent document and the survey were translated into Kinyarwanda and English; English is the official language of instruction at CMHS-UR. Printed informed consent documents were obtained from all participating survey responders. In brief, informed consent detailed the study as a means to better assess cancer management and education needs among Rwandan medical practitioners who provide care for patients diagnosed with cancer. It was explained that all survey data would be kept confidential and that participation in the study was voluntary and without compensation or negative consequences for nonparticipation. Participants were given the opportunity to ask for clarification before providing written consent and were informed that they could exit the study at any time without consequence.

### Statistical Analysis

Summary data for the participant cohort were aggregated to describe demographic and knowledge variables, including age; district/sector of origin; location of DH; and response to true/false questions related to diagnosis, treatment, and referral of breast, oral, and colorectal cancers, among other variables. Categorical variables are presented as frequencies with percentages where appropriate. We used STATA software, version 14.2 (STATA, College Station, TX; Computing Resource Center, Santa Monica, CA) for data management and statistical analysis.

## RESULTS

### Responder Demographics and DH Characteristics

**FIG 1:**
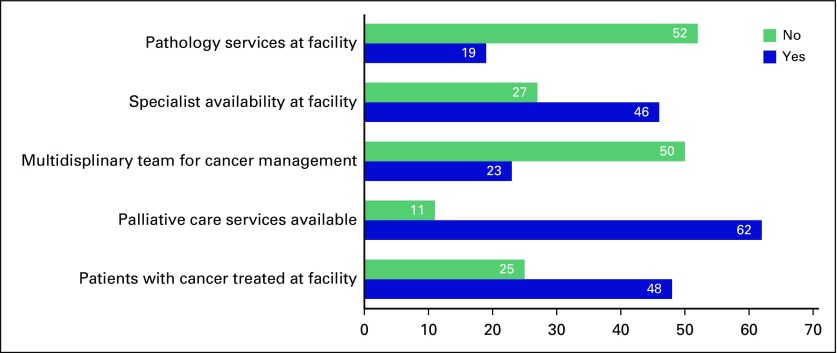
Oncology-related hospital services available to general practitioners.

The overall survey response rate was 76% with 73 of 96 eligible participants electing to complete the survey in its entirety. Consistent with the overall characteristics of GPs in Rwanda, most responders were 25 to 29 years of age (n = 64 [88%]) and male (n = 56 [79%]). Most responders (n = 48 [66%]) worked in facilities where they cared for patients receiving treatment for cancer and palliative care for cancer (n = 62 [85%]). The majority of responders (n = 69 [96%]) reported practicing in facilities in which fewer than five patients with cancer or cancer symptoms are treated per week. The remainder of responder and facility characteristics are listed in Table 1 and Figure 1.

**TABLE 1:**
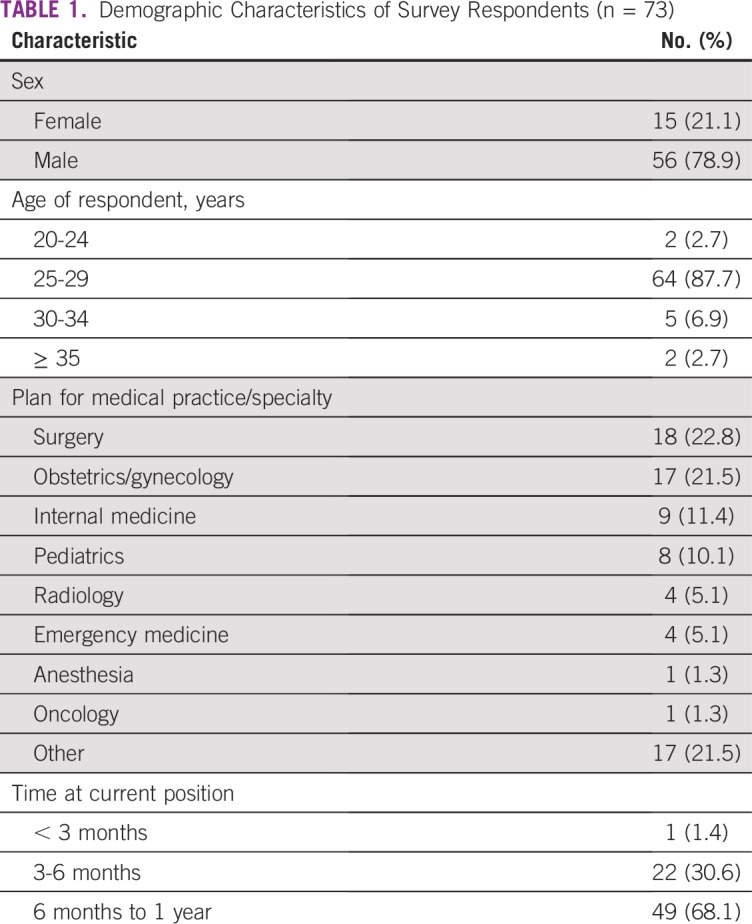
Demographic Characteristics of Survey Respondents (n = 73)

### Educational Gaps at the DH Level

**FIG 2:**
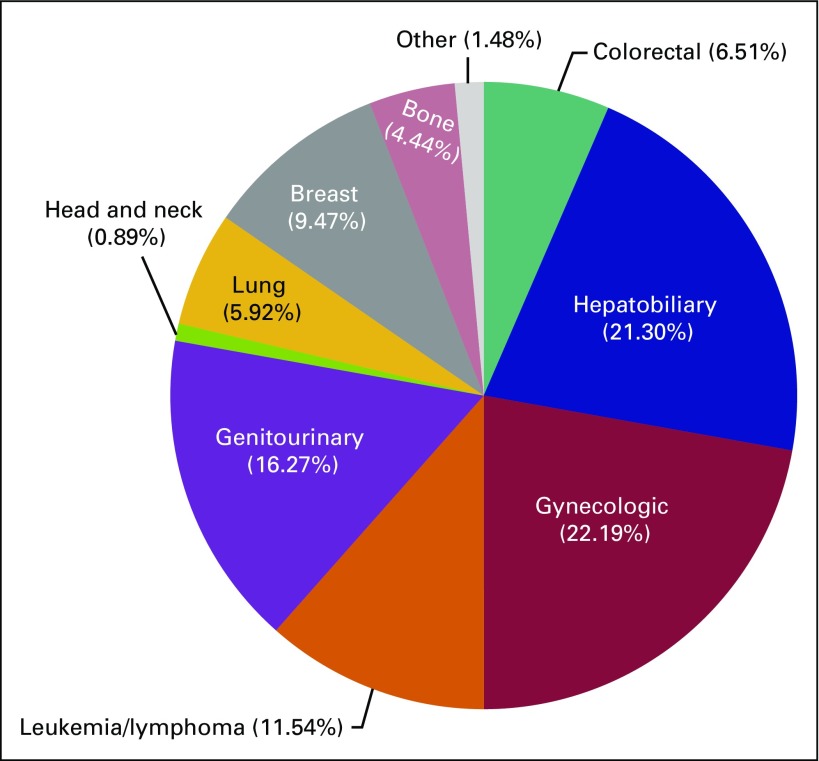
The 383 types of tumors managed at district hospitals in Rwanda. General practitioners were able to choose all cancers among listed options that they encountered and managed during their everyday practice. Answer options included GI cancers (colon, rectal, and stomach), hepatobiliary cancers (pancreas and liver), gynecologic cancers (ovarian, cervical, and endometrial), blood cancers (leukemia and lymphoma), genitourinary cancers (prostate, bladder, penile, prostate, and testicular), oral cancer, lung cancer, breast cancer, bone cancer, and an option to list other cancers.

Educational gaps for GPs at the DH level are listed in Table 2. When asked about their comfort level with taking cancer history and performing physical examination, 19 (20%) of responders were somewhat uncomfortable or gave a neutral response (ie, neither comfortable nor uncomfortable), whereas 54 (74%) responders claimed to be somewhat or very comfortable. However, when asked which physical exam skill responders felt comfortable performing independently, responses varied widely. Although breast and cervical cancer were the most commonly encountered cancers (Fig 2), 64 (88%) respondents were comfortable performing a breast exam but only 10 (14%) were comfortable performing a pelvic exam for cervical lesions. Similarly, whereas 32 (44%) responders were comfortable performing a clinical exam of the head and neck, only 21 (29%) were comfortable doing a visual inspection of the mouth. Only 36 (49%) responders were comfortable performing a digital rectal exam for anorectal or prostatic masses. In terms of undergraduate education and instruction in physical diagnosis skills, more than one half of all responders (n = 53 [72.6%]) desired more instruction during medical school on screening for cervical lesions and performing pelvic exams, 34 (46.6%) desired more instruction performing clinical breast exams, and 31 (42.5%) responders desired more instruction performing digital rectal exams for anorectal or prostatic mass. When asked which cancer-related topics responders wished they had learned about during medical school, the majority (n = 48 [65.8%]) selected instruction on cancer diagnostic guidelines and protocols specific to Rwanda. Other common responses included a desire for more coverage of pain and symptom management (n = 44 [60.3%]) and components of palliative care (n = 43 [58.9%]). The majority of respondents were accessing ancillary educational resources, most commonly UpToDate (Waltham, MA; http://www.uptodate.com; n = 58 [79.5%]), Internet searches (n = 33 [45.2%]), and textbooks (n = 32 [43.8%]).

**TABLE 2:**
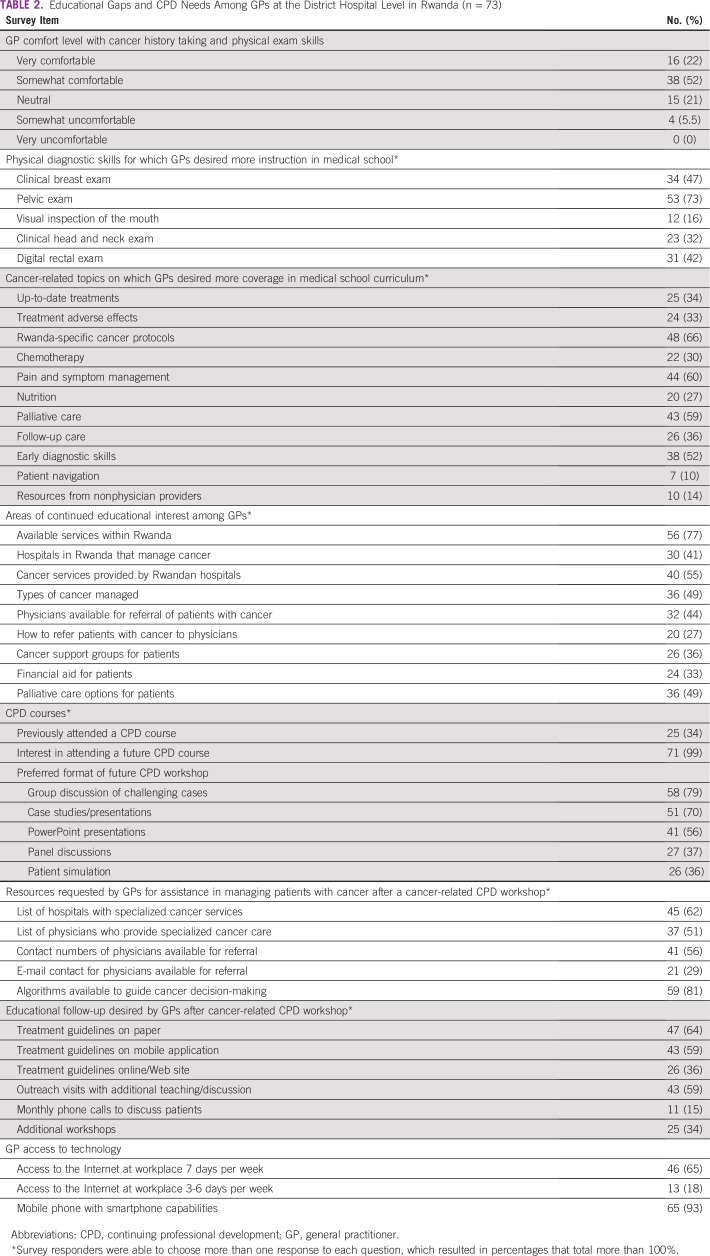
Educational Gaps and CPD Needs Among GPs at the District Hospital Level in Rwanda (n = 73)

### Delays in Care at the DH Level

**FIG 3:**
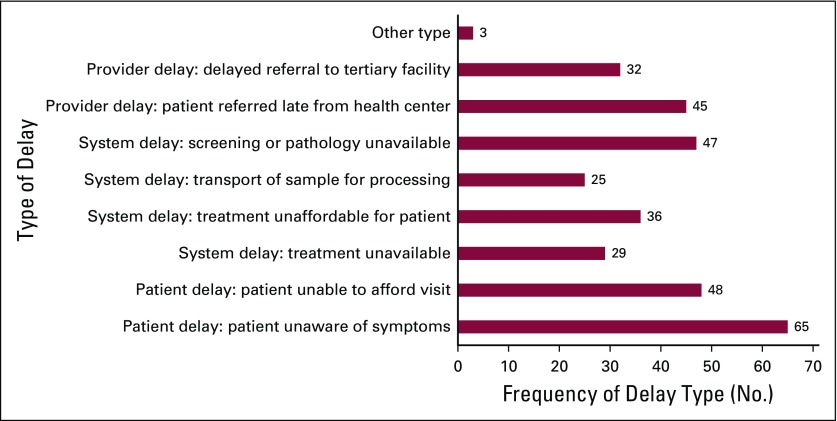
Causes of delay in managing patients with cancer symptoms.

Several questions were included to assess GPs’ perceptions of patient-, provider-, and system-level delays in cancer diagnosis and care. Forty-eight (66%) GPs cited an inability to afford clinic visits and 65 (89%) GPs cited a lack of awareness of symptoms by patients as potential causes of patient-level delays. When asked about reasons for provider-level delays, GPs described inconsistency in the method for referring patients. In Rwanda, patients are referred with a letter and typically present for evaluation to a specialist without a specific appointment with a predetermined physician. The majority of responders (n = 43 [46.7%]) reported using a referral letter directed at a tertiary-care hospital when sending patients for management at a higher or more specialized level of care, whereas only 19 (20%) used a referral letter and a phone number directed primarily at a particular physician. Multiple causes of system-level delays were identified by GPs. Forty-seven (49%) GPs identified a lack of pathology or screening services as possible reasons for a delay in diagnosis, with nearly three quarters (n = 52) of respondents lacking pathology services at their facilities. Several resources commonly used in initial cancer diagnosis or biopsy were found to be limited at the DH level. Only 39% of GPs reported regular availability of breast ultrasound, just 5.8% of GPs reported regular availability of core needle biopsy, and only 4.9% of GPs reported regular availability of colposcopy at their facility. Figure 3 further illustrates potential delays in care as identified by survey responders.

### Future Areas for GP CPD

Fifty-one (69.9%) responders expressed interest in learning more about various areas of cancer management, including diagnostic skills to detect cancers earlier. Forty-eight (65.8 %) expressed interest in learning more about palliative care, and 46 (63.0%) were interested in information on the most up-to-date cancer treatments, including those that were not available in Rwanda. Fifty-six (76.7%) responders also desired more information about which cancer treatment services were available in Rwanda, 40 (54.8%) responders desired information about specific cancer treatment services currently provided by referral facilities in Rwanda, and 36 (49.3%) desired more information about types of cancers managed by referral facilities in Rwanda.

Only 25 (34.3%) responders had ever attended a CPD related to cancer, whereas nearly all respondents (n = 71; [98.6%]) expressed interest in attending a cancer-related CPD. The majority (n = 58 [79.5%]) of respondents preferred to attend a 2-day workshop with a 6-month interval refresher course as opposed to a 1-day workshop with a 6-month refresher course (n = 3 [4.11%]) or a remote learning course (n = 2 [2.74%]). Most respondents (n = 58 [79.5%]) desired to have information at a future workshop presented as an interactive or group discussion of challenging cases, whereas 51 (69.9%) responders would prefer case studies and/or case presentations. Most respondents (n = 59 [80.8%]) desired specific algorithms to help guide cancer diagnosis decision-making, a list of hospitals with specialized cancer services (n = 45 [61.6%]), and contact numbers for physicians available for patient referrals (n = 41 [56.2%]). Most respondents (n = 47 [64.4%]) desired postworkshop educational follow-up in the form of treatment guidelines on paper handouts. Most respondents (n = 59 [80.8%]) had regular access to the Internet at their workplace at least 3 days per week and 65 (89.0%) responders possessed a mobile phone with smart phone capabilities. More detailed information regarding GP desires for CPD course content can be found in Table 2.

## DISCUSSION

Experts predict that by the year 2050, seven of 10 new cancer diagnoses will be among individuals who reside in LMICs.^9^ As cancer rates in LMICs continue to rise, groups such as WHO are advocating for mitigating delays in diagnosis at the provider level via increased diagnostic accuracy and earlier and more appropriate referrals—this starts with education and training primary care providers, such as GPs.^10^ The recent Lancet Commission on Global Oncology called for primary care providers in LMICs taking an expanded role in cancer control.^11,12^ To increase the capacity of generalists to perform in this role, we have chosen to focus on cancer education and professional development of GPs in Rwanda. The current needs assessment is intended to identify educational gaps and identify areas in which care is being delayed at the DH level in Rwanda. Our overall results indicate that first-year GPs practicing at the DH level in Rwanda are limited in both physical examination skills and diagnostic tools for assessing the most common cancers encountered at their health care facilities, with clinical breast and pelvic exams identified as the primary areas of deficiency. Participants were mostly interested in cancer-specific algorithms and guidelines that would aid in caring for patients with cancer in their local clinical setting. Through education and the implementation of algorithms that simplify guidelines for referral of patients with concerning symptoms, we can help to strengthen and streamline referral mechanisms within the country. A program targeting GPs to enhance early diagnosis of cancer can address both provider- and system-level delays.

Results of our needs assessment identified multiple potential areas for improvement in patient-, provider-, and system-level delays that serve as barriers to early cancer diagnosis. The current study identified multiple reasons for delay, including a lack of patient knowledge, inconsistency in referral pathways, and a lack of diagnostic and screening tool availability at the DH level. Our results are consistent with previous studies done in Rwanda. Pace et al^13^ discussed the need to focus on efficient referrals of women with breast cancer as a starting point for promoting earlier diagnosis and decreasing delays in care for patients in Rwanda. Pace et al^14^ also described breast cancer as a problem that affects approximately 55% of patients referred to the Butaro Cancer Center of Excellence with a previously undiagnosed breast concern. The authors demonstrated that most patients with breast cancer at their facility were diagnosed with stage III or IV disease. Data such as these point to the need for more breast diagnostic equipment, such as ultrasound and technicians capable of using these machines, which would allow patients to present earlier in the disease process. Pace’s group also initiated one of the first training modules for nurses at the DH level—a program that was intended to increase nurses’ skill in evaluating common breast complaints and identifying patients in need of referral to the DH. This intervention has also trained district-level clinicians in the evaluation of breast masses.^15^

Our results, however, are not specific to Rwanda but are similar to the experiences described in other LMICs. Otieno and colleagues^16^ reported an average overall time lapse of nearly 90 days between first hospital visit and the initiation of definitive cancer treatment of breast cancer. Onyango and Macharia^17^ also reported significant delays for head and neck cancers, with 56% of patients in their cohort presenting with stage IV disease. In a review of patients with multiple cancer types who presented to a referral hospital in Cameroon, lack of financial support and limited availability in appointment times were cited as top reasons for delays before the first appointment.^18^ Authors in Nepal identified delays in the diagnosis of cervical cancer related to multiple steps along the referral pathway, including patient delay and health care provider delay.^19^

The current study has several important limitations. First, GPs surveyed were all young and in their first year of practice. Surveying this population allowed for a timely assessment of the recent medical school curriculum but may bias our sample toward practitioners who are less experienced at the DH level. Future assessments can include broader coverage of practitioners with a wider range of clinical experience. More pressing is the need to objectively evaluate the medical school curriculum to ensure that the curriculum matches the practical needs of the trainee once he or she graduates. Second, availability of both practitioners and services at the district level likely varies widely, possibly even on a day-to-day basis. It can be difficult or impossible to predict when a particular material supply may go into shortage or when a particular health care provider leaves his or her practice at the district, making the services of that provider no longer available. Future interventions may allow for an electronic database of district-level supplies and providers that can be updated from any location; however, the financial and personnel support for such a database is not currently possible given the limited resources. Finally, the feasibility of educating GPs in the identification and triage of patients who present with cancer-concerning symptoms may be a challenge given the disparate geographic location of the GPs; however, as Rwanda is a small country and refresher courses for GPs are routinely organized, this type of training may be bundled with the currently existing system for continued medical education for GPs or can be integrated earlier into the medical school curriculum.

This study establishes the necessary components for a program intended to mitigate delays in GP referrals of patients with common cancers in Rwanda. An early diagnosis training program targeting GPs should be developed that integrates the findings of this study. Strengthening the current health care system through cancer-related CPDs for Rwanda’s front-line providers should improve recognition of early cancer symptoms. Results of this study may also help identify and develop a pathway for referral that is sustainable and equips front-line health providers with the knowledge and skills necessary to aid in earlier detection of these cancers.

Despite being the gatekeepers and main health care providers for a majority of patients, GPs who practice at the DH level in Rwanda are limited in both physical examination skills and diagnostic tools for assessing the most common cancers encountered at their health care facilities. CPD for GPs in Rwanda should be a core component of national cancer control plans to improve accurate and timely diagnosis of cancer. Responders desire future cancer education–related CPDs with algorithms to help guide clinical decision-making for patients with cancer symptoms at the DH level appropriate to the cancer management resources currently available in Rwanda.
